# hs-CRP/HDL-C can predict the risk of all cause mortality in cardiovascular-kidney-metabolic syndrome stage 1-4 patients

**DOI:** 10.3389/fendo.2025.1552219

**Published:** 2025-04-10

**Authors:** Fengjiao Han, Haiyang Guo, Hao Zhang, Yang Zheng

**Affiliations:** First Affiliated Hospital of Jilin University, Changchun, China

**Keywords:** cardiovascular-kidney-metabolic syndrome, high-sensitivity C-reactive protein, high-density lipoprotein cholesterol, mortality, CHARLS

## Abstract

**Background:**

The precise function of the hs-CRP/HDL-C ratio in forecasting the long-term mortality risk of patients with stages 1-4 of Cardiovascular-Kidney-Metabolic (CKM) syndrome remains inadequately delineated. This study investigates the potential correlation between the hs-CRP/HDL-C ratio and long-term mortality risk in individuals with CKM syndrome stages 1-4.

**Methods:**

This prospective cohort study utilises data from the China Health and Retirement Longitudinal Study (CHARLS) project, encompassing 6,719 people who satisfied stringent criteria. We developed three Cox proportional hazards regression models to investigate the potential relationship between the hs-CRP/HDL-C ratio and long-term mortality risk in patients with CKM stages 1-4. We employed Restricted Cubic Spline (RCS) curves for analysis to identify any potential nonlinear correlations. Furthermore, we performed Receiver Operating Characteristic (ROC) curve analysis to evaluate predictive performance and identify the appropriate cut-off value. To enhance the research findings, we conducted a stratified analysis to investigate the influence of various sociodemographic factors on this association.

**Results:**

In individuals with CKM syndrome stages 1-4, the 10-year incidence of all-cause mortality was 14.1%. Upon controlling for additional potential confounding variables, the outcomes of the Cox proportional hazards regression model distinctly demonstrated a statistically significant linear positive association between the hs-CRP/HDL-C ratio and the long-term mortality risk in patients. For each quartile increase in the hs-CRP/HDL-C ratio, the probability of poor outcomes (i.e., mortality) escalated by 15% (Hazard Ratio, HR = 1.15, 95% Confidence Interval, CI: 1.09–1.22, p-value < 0.001). Moreover, the integration of the hs-CRP/HDL-C ratio into the baseline risk prediction model, with all pertinent factors thoroughly adjusted, markedly enhanced the model’s predictive capacity, facilitating a more precise assessment of long-term mortality risk in patients with CKM syndrome stages 1-4.

**Conclusion:**

This study identified a positive linear association between the hs-CRP/HDL-C ratio and long-term mortality risk in patients with stages 1 to 4 of CKM syndrome. This remarkable discovery not only offers a crucial reference for enhancing early individualised treatment options but also greatly aids in the early identification of patients with poor prognoses, hence presenting a novel perspective for improving clinical management pathways for these individuals.

## Introduction

The American Heart Association (AHA) President’s Council Bulletin published in October 2023 formally designated Cardiovascular-Kidney-Metabolic (CKM) syndrome as a systemic and progressive illness state. The essence resides in the intricate pathophysiological connections involving metabolic risk factors, chronic kidney disease (CKD), and the cardiovascular system, including mechanisms including inflammation, oxidative stress, and insulin resistance. Over time, these degenerative processes interact synergistically, facilitating the progression of atherosclerosis while inducing cardiac structural remodelling and a slow deterioration of renal function, so markedly elevating the mortality risk for patients (1, 2). Research indicates that around 5.3% of the adult population in the United States experiences concurrent cardiac, kidney, and metabolic disorders, a demographic that typically receives insufficient treatment, hence representing a burgeoning health issue (3).

Dyslipidaemia is a primary factor in the onset and advancement of Cardiovascular-Kidney-Metabolic (CKM) syndrome. Multiple studies have established that triglyceride (TG) and low-density lipoprotein cholesterol (LDL-C) levels serve as significant predictors for the risk and prognosis of cardiovascular diseases (CVD) (4–6). Furthermore, the triglyceride-glucose (TyG) index and the estimated glucose disposal rate (eGDR) have also been shown to increase the risk of cardiovascular diseases in patients with CKM syndrome stages 0-3, subsequently elevating their risk of mortality (7, 8). In contrast, high-density lipoprotein cholesterol (HDL-C) has been conventionally viewed as a contributor to cholesterol efflux, endothelial protection, and antioxidant functions, with its levels negatively correlated to the onset of cardiovascular disease (CVD) (9, 10). A study published in the American Journal of Kidney Diseases presents a paradox: In chronic kidney disease (CKD), a significant decline in the activity of key high-density lipoprotein (HDL)-related enzymes such as paraoxonase 1 (PON1), nitric oxide (NO) synthase (NOS), and LCAT results in a reduction of HDL-C’s functional activity, subsequently transitioning it into a proinflammatory state. This state not only accelerates the oxidation of phospholipids but also promotes the accumulation of serum amyloid A and C-reactive protein (CRP), thereby impairing HDL’s innate protective role against atherosclerosis. This series of changes ultimately exacerbates the abnormal efflux of cholesterol from cells, further driving the progression of the disease (11–15). Recent studies have shown that the Atherogenic Index of Plasma (AIP), calculated through the logarithmic ratio of triglycerides (TG) to high-density lipoprotein cholesterol (HDL-C), can facilitate the progression of patients with CKM syndrome from stages 0-3 to stage 4 (16). Furthermore, research has found that the risk of mortality in stage 4 of CKM syndrome is significantly higher than that in stages 0-3 (17). Inflammatory indicators, including neutrophil count and high-sensitivity C-reactive protein (hs-CRP) levels, have demonstrated predictive value for long-term prognosis in cardiovascular disease patients (18, 19). Additionally, monocyte and lymphocyte counts have also been found to predict mortality risk in CKD patients (20). Previous studies have delved into the roles of inflammation, insulin resistance, and dyslipidemia in the pathophysiological processes of CKM (21–23). Overall, high levels of inflammatory markers and dyslipidemia are closely associated with renal function deterioration and adverse cardiac prognosis in CKD patients (5, 24).

Given the close correlation between inflammation and lipid metabolism, numerous studies have integrated inflammatory and metabolic indicators for comprehensive consideration. Prior studies have demonstrated that the monocyte to high-density lipoprotein cholesterol (HDL-C) ratio and the white blood cell count to HDL-C ratio can reliably forecast mortality in individuals with coronary artery disease (25, 26). Additionally, the ratio of monocytes to HDL-C is also closely associated with the incidence of cardiorenal syndrome (27). The ratio of high-sensitivity C-reactive protein (hs-CRP) to HDL-C, known as the hs-CRP/HDL-C ratio, has demonstrated prognostic significance for cardiovascular illnesses in middle-aged and elderly populations (28).

However, despite these studies revealing the significant roles of inflammatory and lipid metabolic indicators in cardiovascular diseases, research on the specific association between the hs-CRP/HDL-C ratio and all-cause mortality in patients with Cardiovascular-Kidney-Metabolic (CKM) syndrome stages 1-4 remains inadequate. Therefore, this study aims to deeply analyze the relationship between the hs-CRP/HDL-C ratio and all-cause mortality in patients with CKM stages 1-4, in order to provide new insights and strategies for improving long-term prognosis in this patient population.

## Materials and methods

### Study population and data source

The China Health and Retirement Longitudinal Study (CHARLS) is a nationally representative longitudinal survey. The nationwide baseline survey of CHARLS was executed from June 2011 to March 2012, with participants being monitored biennially via face-to-face Computer-Assisted Personal Interviews (CAPI). Physical assessments and blood samples are obtained during each biennial follow-up (29). The CHARLS data collection received approval from the Peking University Biomedical Ethics Review Committee (IRB00001052-11015), and the research protocol complied with the ethical criteria of the 1975 Helsinki Declaration. All participants in the study granted their consent subsequent to obtaining comprehensive written information.

The flowchart (**Supplementary Figure S1**) describes the inclusion and exclusion criteria for this study. Initially, there were 17,708 individual participants in the CHARLS baseline survey. We excluded 5,861 participants who lacked blood samples. Additionally, we excluded participants missing key data such as age, height, weight, gender, hs-CRP, HDL-C, as well as those who were lost to follow-up (non-deceased) during the 2011-2020 follow-up period and those with cancer at baseline, totaling 3,562 exclusions. The remaining 8,285 participants were staged for CKM, and 511 participants in stage 0 were also excluded, leaving 7,466 participants. Further exclusions were made for participants with hs-CRP/HDL-C values in the top 5% and bottom 5%. Therefore, a total of 6,719 participants with CKM stages 1-4 were included in the analysis.

### Variables

#### Calculation of hs-CRP/HDL-C ratio

In this study, the hs-CRP/HDL-C ratio was calculated by dividing the hs-CRP level (mg/L) by the HDL-C level (mg/dL/1000) (28). Participants were divided into four groups based on the quartiles of the hs-CRP/HDL-C ratio: Group 1 (Q1) with hs-CRP/HDL-C values ranging from 4.84 to 11.59; Group 2 (Q2) with hs-CRP/HDL-C values ranging from 11.59 to 22.27; Group 3 (Q3) with hs-CRP/HDL-C values ranging from 22.27 to 44.81; and Group 4 (Q4) with hs-CRP/HDL-C values ranging from 44.81 to 219.

#### Definition of CKM syndrome stage to 4

The staging of CKM syndrome ranges from 1 to 4, classified according to the American Heart Association (AHA) Presidential Advisory on Cardiovascular Disease and Stroke in Patients with Cardiovascular-Kidney-Metabolic (CKM) Syndrome (1). The stages are delineated as follows: Stage 1 is characterised by overweight/obesity, abdominal obesity, or dysfunctional adipose tissue in the absence of other metabolic risk factors or chronic kidney disease (CKD); Stage 2 involves individuals exhibiting metabolic risk factors (hypertriglyceridemia [≥135 mg/dL], hypertension, metabolic syndrome, diabetes) or CKD; Stage 3 encompasses subclinical cardiovascular disease (CVD), with subclinical CVD risk equivalents indicated by a high 10-year CVD risk in very high-risk CKD (CKD stages G4 or G5 or classified as very high risk by KDIGO); Stage 4 pertains to individuals with excessive/dysfunctional obesity, additional CKD risk factors, or clinical CVD in the context of CKD. The Framingham Risk Score’s prediction of a high 10-year cardiovascular disease risk functions as a risk equivalent for subclinical cardiovascular disease (30). The estimated glomerular filtration rate (eGFR) is computed via the Chinese Modification of Diet in Renal Disease (C-MDRD) equation (31) and classified into CKD stages according to Kidney Disease Improving Global Outcomes (KDIGO) (1).

### Follow-up endpoint

The principal endpoint of this trial is defined as all-cause mortality across the 10-year follow-up period, ascertained from the interview status (i.e., alive or died) of participants in waves 2, 3, 4, 5, and 6. Detailed information regarding interview dates is accessible from all three follow-up stages; however, precise data on time of death is exclusively available in waves 2 and 6. Survival time is defined as the duration from the baseline survey date to the date of the participant’s death, provided that the death event has been adequately documented. In instances where precise mortality data is unavailable, we have conducted a reasonable calculation of survival duration based on the median interval between the first interview date and the wave in which mortality information was documented.

### Data collection

For this study, we systematically gathered the following data: demographic details including age, gender, educational attainment, and marital status; physiological measurements such as systolic blood pressure (SBP), diastolic blood pressure (DBP), height, weight, and waist circumference; lifestyle factors regarding smoking and alcohol consumption; and medical histories of hypertension, diabetes, dyslipidaemia, cancer, among others. Furthermore, biochemical markers such as glycated haemoglobin A1c (HbA1C), fasting blood glucose (FBG), triglycerides (TG), total cholesterol (TC), high-density lipoprotein cholesterol (HDL-c), low-density lipoprotein cholesterol (LDL-c), blood urea nitrogen (BUN), serum creatinine (Scr), high-sensitivity C-reactive protein (hs-CRP), and uric acid (UA) were evaluated.

Hypertension was defined as follows: participants were classified as hypertensive if they had a history of hypertension, were undergoing particular treatment for hypertension, or had a systolic blood pressure (SBP) ≥140 mmHg or a diastolic blood pressure (DBP) ≥90 mmHg at baseline (32). The diagnostic criteria for diabetes included participants reporting a history of diabetes or being treated for diabetes, or having an FBG ≥7.0 mmol/L (126 mg/dL) or HbA1C ≥6.5% at baseline (33). Dyslipidemia was determined based on participants reporting a history of dyslipidemia or having any one of the following: TG >2.26 mmol/L, TC >6.22 mmol/L, LDL-c >4.14 mmol/L, or HDL-c <1.04 mmol/L. Other medical conditions were determined based on participants’ self-reports.

## Statistical analysis

The participants in this study were divided into four groups (Q1-Q4) based on the quartiles of the hs-CRP/HDL-C ratio. For continuous variables showing normal distribution, statistical data were described using the mean and standard deviation, and analysis of variance (ANOVA) was used to infer differences between groups. For continuous variables not following a normal distribution, statistical descriptions were made using the median and interquartile range, and the Kruskal-Wallis H test was used to examine differences between groups. Categorical variables were characterized by frequency and percentage, and the χ² test was used to assess differences between groups. Kaplan-Meier curves were employed to display mortality rates across different groups, and a Cox proportional hazards regression model was utilized to explore the association between the hs-CRP/HDL-C ratio and all-cause mortality, with hazard ratios (HRs) and 95% confidence intervals (CIs) for patients with stages 1-4 CKM. Restricted cubic spline regression for HRs was used to investigate the potential nonlinear relationship between the hs-CRP/HDL-C ratio and all-cause mortality in patients with stages 1-4 CKM. Additionally, we investigated whether the hs-CRP/HDL-C ratio could improve the predictive performance of a baseline risk model, which included age, gender, education level, marital status, current smoking status, diabetes mellitus (DM), body mass index (BMI), fasting blood glucose, and blood uric acid. Various measures were used to assess the incremental predictive performance of all-cause mortality risk after introducing the hs-CRP/HDL-C ratio into the baseline risk model, including the calculation of the C-statistic, continuous net reclassification improvement (NRI), and integrated discrimination improvement (IDI). The C-statistic was calculated to represent the performance of each model using the “Survival” R package. Both continuous NRI and IDI were calculated using the “survIDINRI” R package. Finally, to explore the association between the hs-CRP/HDL-C ratio and all-cause mortality in patients with stages 1-4 CKM across different demographic characteristics, subgroup analyses and interaction analyses were conducted for age, smoking status, education level, diabetes, metabolic syndrome, and CKM stage. All statistical analyses were performed using R software (version 4.4.1), and a two-sided P-value < 0.05 was considered statistically significant.

## Results

### Baseline characteristics

**Figure 1:**
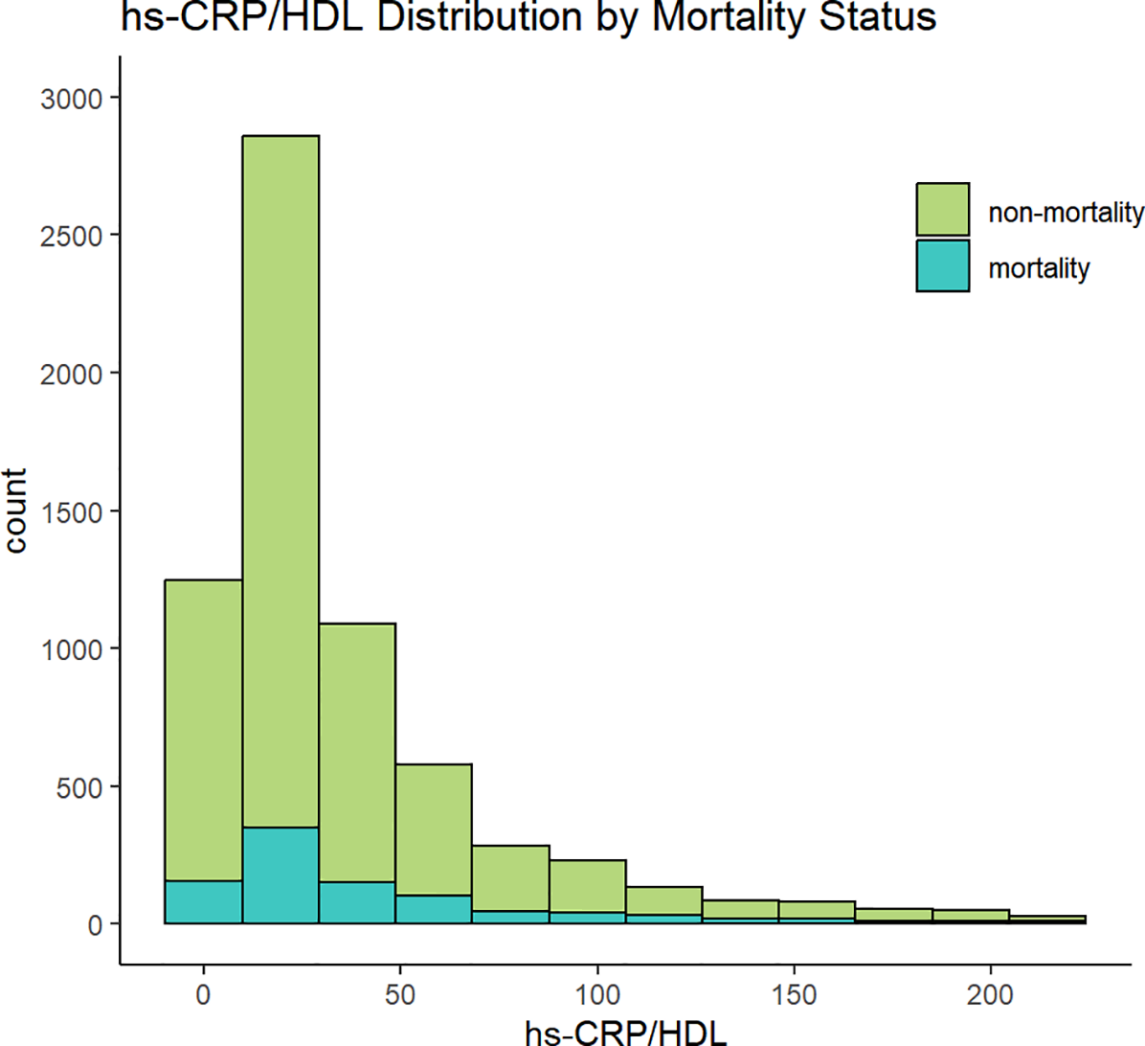
Distribution of hs-CRP/HDL.

**Figure 2:**
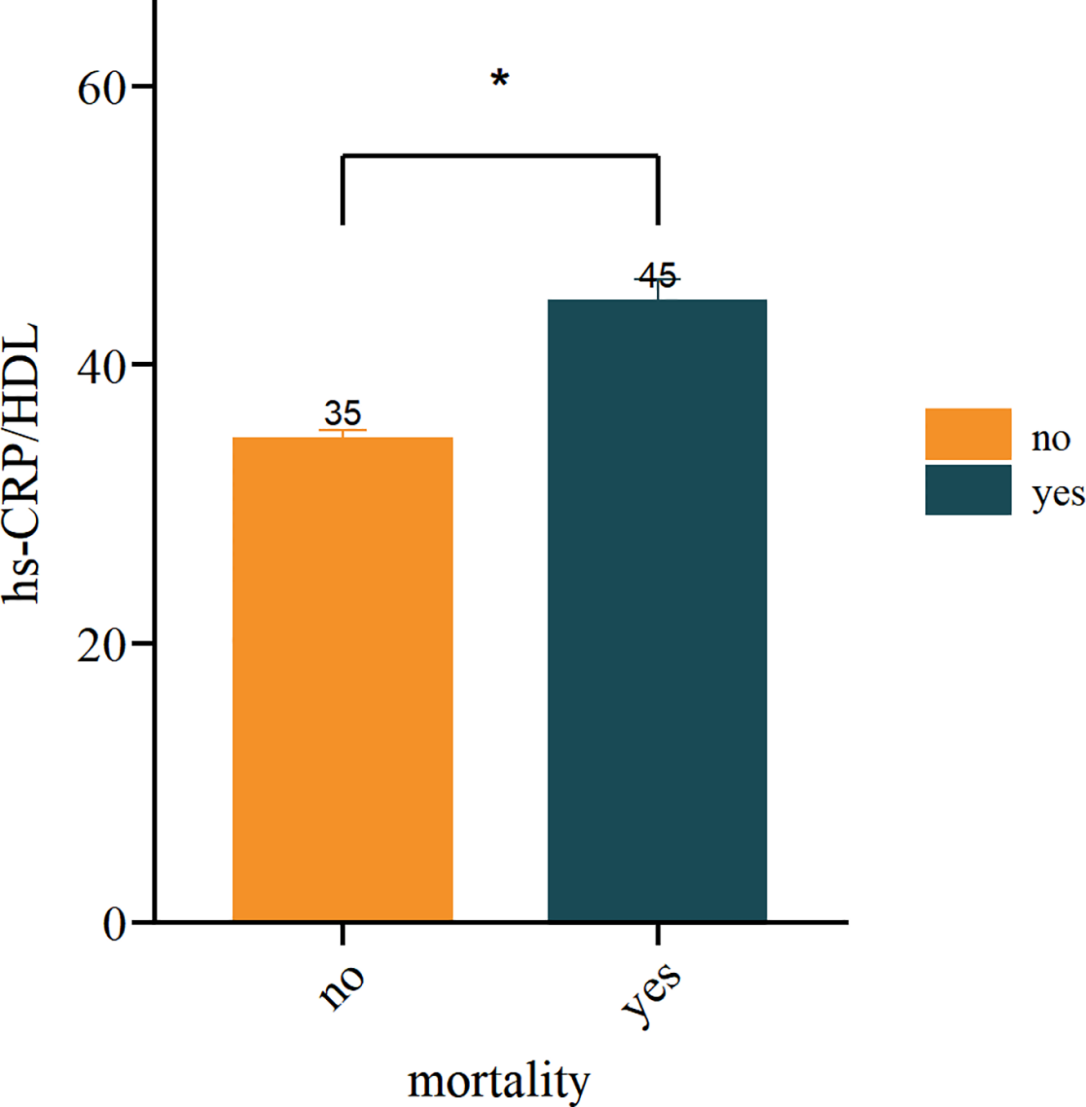
Analysis of inter group differences in hs-CRP/HDL values between mortality and non mortality groups. (*indicates p < 0.05).

This study comprised a total of 6,719 participants from CHARLS. **Table 1** delineates the baseline characteristics of the enrolled participants: the mean age was 59 years, with 52.5% identifying as female and 47.5% as male. Upon categorisation by the quartiles of the hs-CRP/HDL-C ratio, we observed that persons in the higher hs-CRP/HDL-C ratio groups exhibited increased proportions of hypertension, dyslipidaemia, diabetes mellitus, cardiovascular disease, metabolic syndrome, as well as elevated rates of smoking and alcohol consumption (P < 0.05). Moreover, members of these groups demonstrated elevated levels of BMI, waist circumference, glycosylated haemoglobin, fasting blood glucose, total cholesterol, creatinine, uric acid, low-density lipoprotein cholesterol, and high-sensitivity C-reactive protein, alongside diminished levels of high-density lipoprotein cholesterol and eGFR, with statistically significant differences (P < 0.05). No significant differences were seen among the four groups for gender, education level, marital status, family residence, and the existence of CKD (P-values > 0.05). **Figure 1** illustrates the distribution of the hs-CRP/HDL-C ratio alongside the mortality rate, with a median value of 22.27. The Kolmogorov-Smirnov (K-S) test yielded a p-value < 0.05, signifying that the hs-CRP/HDL-C ratio has a skewed distribution. **Figure 2** illustrates the comparison of hs-CRP/HDL-C ratios between deceased and non-deceased participants, revealing that deceased participants exhibited elevated hs-CRP/HDL-C values compared to their non-deceased counterparts, with a statistically significant difference.

**Table 1:** Baseline characteristics of individuals classified by hs-CRP/HDL quartiles.

Variable	Level	Q1 (4.84-11.56)	Q2 (11.56-22.27)	Q3 (22.27-44.81)	Q4 (44.81-219)	p
n		1679	1680	1679	1680	
Age,year		58.77 (9.56)	59.32 (9.55)	59.88 (9.44)	60.64 (9.90)	<0.001
Gender,n (%)	Female	907 (54.0)	882 (52.5)	866 (51.6)	872 (51.9)	0.500
	Male	772 (46.0)	798 (47.5)	813 (48.4)	808 (48.1)	
Education,n (%)	primary or below	1532 (91.2)	1536 (91.4)	1514 (90.2)	1532 (91.2)	0.615
	second/high school or above	147 (8.8)	144 (8.6)	164 (9.8)	148 (8.8)	
Marital,n (%)	married	1481 (88.2)	1485 (88.4)	1470 (87.6)	1447 (86.1)	0.182
	unmarried	198 (11.8)	195 (11.6)	209 (12.4)	233 (13.9)	
HTN,n (%)	no	1061 (63.6)	1020 (60.9)	899 (53.6)	785 (46.9)	<0.001
	yes	608 (36.4)	654 (39.1)	779 (46.4)	888 (53.1)	
DM,n (%)	no	1500 (89.7)	1460 (87.1)	1387 (82.8)	1310 (78.2)	<0.001
	yes	173 (10.3)	216 (12.9)	289 (17.2)	366 (21.8)	
DL,n (%)	no	1159 (69.8)	970 (58.3)	786 (47.3)	649 (38.9)	<0.001
	yes	502 (30.2)	693 (41.7)	874 (52.7)	1021 (61.1)	
CKD,n (%)	no	1544 (92.5)	1564 (93.3)	1562 (93.4)	1577 (94.0)	0.405
	yes	125 (7.5)	113 (6.7)	111 (6.6)	101 (6.0)	
CVD,n (%)	no	1473 (87.7)	1460 (86.9)	1427 (85.0)	1401 (83.4)	0.001
	yes	206 (12.3)	220 (13.1)	252 (15.0)	279 (16.6)	
MetS,n (%)	no	1544 (92.3)	1397 (83.2)	1246 (74.6)	1071 (64.0)	<0.001
	yes	129 (7.7)	282 (16.8)	425 (25.4)	603 (36.0)	
Take medicine for diabetes,n (%)	no	1640 (97.7)	1628 (96.9)	1616 (96.2)	1583 (94.2)	<0.001
	yes	39 (2.3)	52 (3.1)	63 (3.8)	97 (5.8)	
Take medicine for hypertension,n (%)	no	1458 (86.8)	1389 (82.7)	1286 (76.6)	1194 (71.1)	<0.001
	yes	221 (13.2)	291 (17.3)	393 (23.4)	486 (28.9)	
Take medicine for dyslipidemia,n (%)	no	1662 (99.0)	1665 (99.1)	1657 (98.7)	1654 (98.5)	0.291
	yes	17 (1.0)	15 (0.9)	22 (1.3)	26 (1.5)	
Lives in rural or urban,n (%)	Urban Community	495 (29.5)	516 (30.7)	565 (33.7)	616 (36.7)	<0.001
	Rural Village	1184 (70.5)	1164 (69.3)	1114 (66.3)	1064 (63.3)	
Annual household income,RMB		21002.7 (30853.5)	21102.7 (29291.8)	21304.5 (45703.2)	22106.9 (33644.6)	0.882
Mortality,n (%)	no	1476 (87.9)	1471 (87.6)	1454 (86.6)	1369 (81.5)	<0.001
	yes	203 (12.1)	209 (12.4)	225 (13.4)	311 (18.5)	
Smoking,n (%)	Ex-smoker	114 (6.8)	146 (8.7)	148 (8.8)	191 (11.4)	0.001
	Non-smoker	1025 (61.2)	1005 (59.8)	994 (59.2)	976 (58.2)	
	smoker	537 (32.0)	529 (31.5)	537 (32.0)	511 (30.5)	
Drinking,n (%)	no	1061 (63.2)	1095 (65.2)	1158 (69.0)	1169 (69.6)	<0.001
	yes	618 (36.8)	585 (34.8)	521 (31.0)	511 (30.4)	
eGFR		107.02 (26.11)	105.74 (26.97)	103.03 (27.49)	102.13 (31.81)	<0.001
BMI, kg/m2		22.56 (3.31)	23.54 (3.70)	24.21 (3.72)	24.79 (4.21)	<0.001
Waist measurement,cm		81.75 (11.07)	84.13 (11.77)	86.65 (11.70)	88.32 (13.06)	<0.001
Sbp,mmHg		128.82 (21.22)	130.20 (20.77)	132.46 (21.63)	135.22 (22.21)	<0.001
Dbp,mmHg		74.73 (12.10)	75.65 (11.86)	76.68 (12.42)	77.94 (12.11)	<0.001
Glycated hemoglobin,mg/dl		5.16 (0.68)	5.24 (0.73)	5.32 (0.87)	5.42 (0.99)	<0.001
Glucose,mg/dl		104.84 (25.24)	108.37 (30.16)	112.61 (40.18)	118.11 (47.88)	<0.001
TG,mg/dl		105.19 (57.88)	126.18 (85.95)	148.28 (110.60)	170.33 (154.17)	<0.001
TC,mg/dl		194.58 (36.19)	193.42 (37.70)	197.79 (40.03)	194.69 (41.49)	0.009
LDL-C,mg/dl		117.37 (32.51)	118.07 (34.00)	120.13 (36.38)	115.76 (38.15)	0.004
Creatinine,mg/dl		0.76 (0.18)	0.77 (0.18)	0.80 (0.26)	0.81 (0.34)	<0.001
Uric acid,mg/dl		4.19 (1.18)	4.39 (1.21)	4.58 (1.23)	4.76 (1.31)	<0.001
hs-CRP,mg/L		0.47 (0.15)	0.84 (0.26)	1.50 (0.51)	3.83 (2.08)	<0.001
HDL-C,mg/dl		58.06 (14.51)	51.51 (13.51)	47.91 (13.56)	43.85 (13.10)	<0.001

Data were expressed as mean  ( SD) or n (%).HTN hypertension, DM diabetes, DL dyslipidemia, CKD chronic Kidney Disease, CVD cardiovascular disease, Mets metabolic Syndrome, eGFR Estimated Glomerular Filtration Rate, BMI body mass index, Sbp systolic blood pressure, Dbp diastolic pressure, TG triglyceride, TC total cholesterol, LDL-C low-density lipoprotein cholesterol, hs-CRP hypersensitive C-Reactive Protein, HDL-C high-density lipoprotein cholesterol.

### The relationship between the hs-CRP/HDL-C ratio and all-cause mortality among participants with CKM stages 1-4

**Figure 3:**
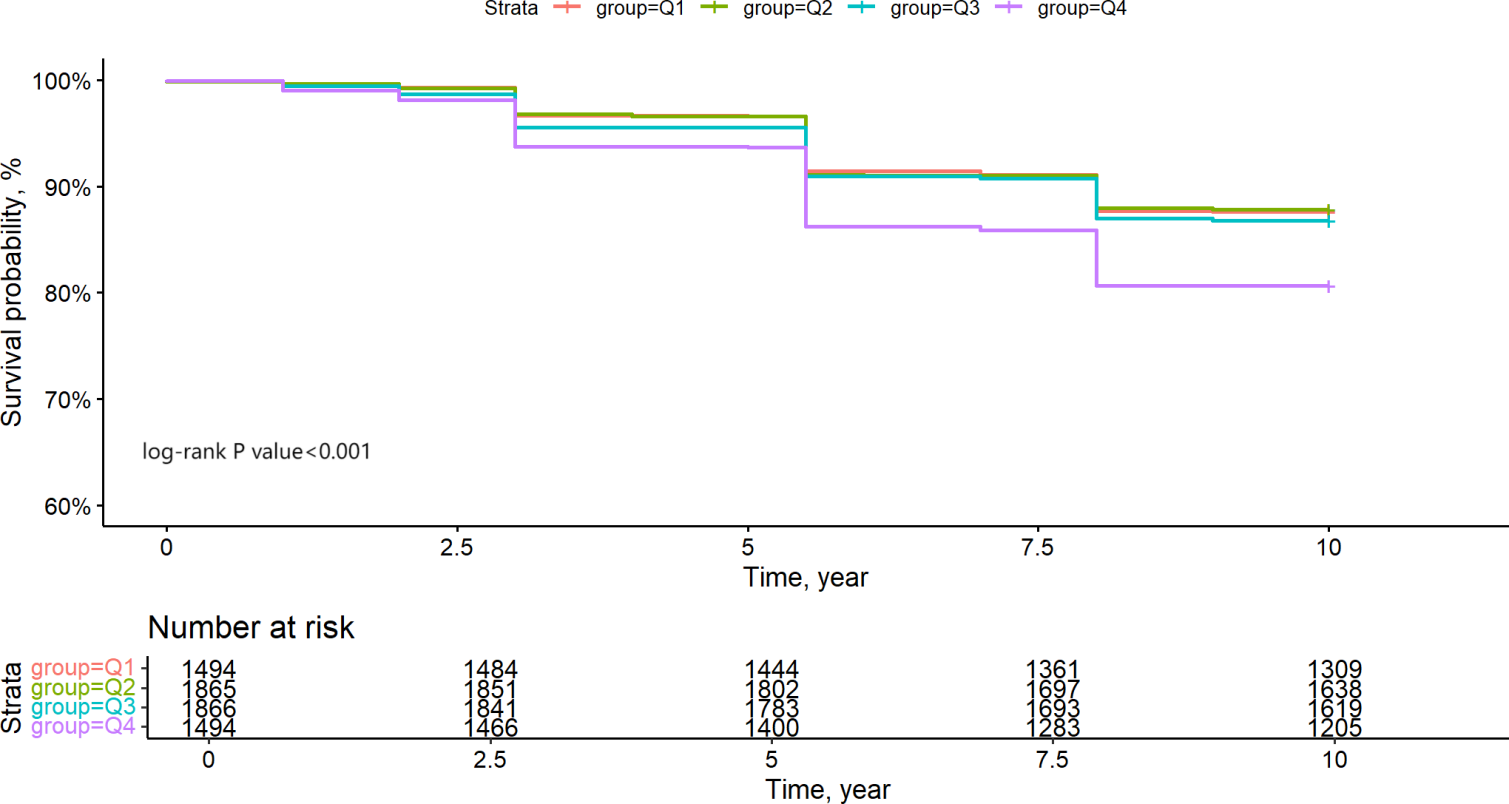
Kapla-Meier estimates 10-year survival rate of CKM1-4 participants based on hs-CRP/HDL quartile grouping.

Initially, we constructed Kaplan-Meier survival curves to illustrate the all-cause mortality risk among various groups. **Figure 3** illustrates that patients in the Q4 group had markedly lower survival rates than the other three groups (Log-rank test P-value < 0.001), although no significant variations in survival rates were detected among the Q1, Q2, and Q3 groups. The comparison of P values between groups is shown in **Supplementary Table S1**. We additionally re-included participants with hs-CRP/HDL-C ratios in the top 5% and bottom 5% (totalling 7,466 individuals) for Kaplan-Meier analysis, revealing no significant alterations, hence affirming the stability of our findings (**Supplementary Figure S2**).

In the 10-year follow-up period (2011-2020), there were 948 fatalities among 6719 individuals with stages 1-4 CKM, yielding an all-cause mortality rate of 14.1%. Three Cox proportional hazards models were designed to precisely evaluate the relationship between the hs-CRP/HDL-C ratio and all-cause mortality in patients with stages 1-4 CKM. Model 1 was adjusted solely for age and gender; Model 2 included adjustments for age, gender, marital status, educational attainment, BMI, smoking, and alcohol consumption, annual household income and lives in rural or urban; Model 3 represented the fully adjusted model, encompassing all potential confounding variables, namely age, gender, marital status, educational attainment, BMI, smoking, alcohol consumption, annual household income, lives in rural or urban, diabetes, hypertension, triglycerides, medication history, fasting blood glucose, low-density lipoprotein cholesterol, uric acid, total cholesterol, and blood creatinine. **Table 2** demonstrates that in the fully adjusted model, each quartile increase in the hs-CRP/HDL-C ratio correlated with a 15% elevation in mortality risk (HR = 1.15, 95% CI: 1.09–1.22, p<0.001). To clarify the association between the hs-CRP/HDL-C ratio and all-cause mortality in individuals with CKM stages 1-4, the hs-CRP/HDL-C ratio was categorized into quartiles. In the comprehensively adjusted Model 3, participants in the fourth quartile (Q4) of the hs-CRP/HDL-C ratio exhibited a hazard ratio (HR) of 1.55 (95% CI: 1.24–1.93, P<0.001) when compared to those in the first quartile (Q1), signifying a 55% elevated risk of all-cause mortality for Q4 participants relative to Q1 participants, with a statistically significant distinction. Nonetheless, in all three models, no statistically significant changes in all-cause mortality risk were observed between individuals in the second quartile (Q2) and third quartile (Q3) as compared to those in the first quartile (Q1) (P>0.05). We re-included participants with hs-CRP/HDL ratios in the top 5% and bottom 5% (totaling 7,466 individuals) for COX regression analysis and observed no significant alterations in the risk estimates, so affirming the robustness of our findings (**Supplementary Table S2**).

**Table 2:** The correlation between hs-CRP/HDL and ten-year mortality risk in CKM syndrome stage 1-4 population.

Variables	Model 1		Model 2		Model 3	
	HR (95%)	P value	HR (95%)	P value	HR (95%)	P value
hs-CRP/HDL per IQR	1.13 (1.08,1.19)	<0.001	1.16 (1.10,1.22)	<0.001	1.15 (1.09,1.22)	<0.001
hs-CRP/HDL quartile						
Q1	ref		ref		ref	
Q2	0.99 (0.81,1.20)	0.90	1.05 (0.84,1.32)	0.65	1.06 (0.84,1.34)	0.63
Q3	1.05 (0.87,1.28)	0.58	1.19 (0.95,1.49)	0.12	1.17 (0.93,1.47)	0.18
Q4	1.40 (1.17,1.67)	<0.001	1.61 (1.30,1.99)	<0.001	1.55 (1.24,1.93)	<0.001

**Table 2** The correlation between hs-CRP/HDL and ten-year mortality risk in CKM syndrome stage 1-4 population.

Model 1: adjusted age, gender.

Model 2: adjusted age, gender, marital, bmi, smoking, drinking, education, Annual household income and Lives in rural or urban.

Model 3: age, gender, marital, bmi, smoking, drinking, education, Annual household income, Lives in rural or urban, DM, HTN, medication history, triglycerides, glucose, ldl cholesterol, uric acid, total cholesterol and creatinine.

**Figure 4:**
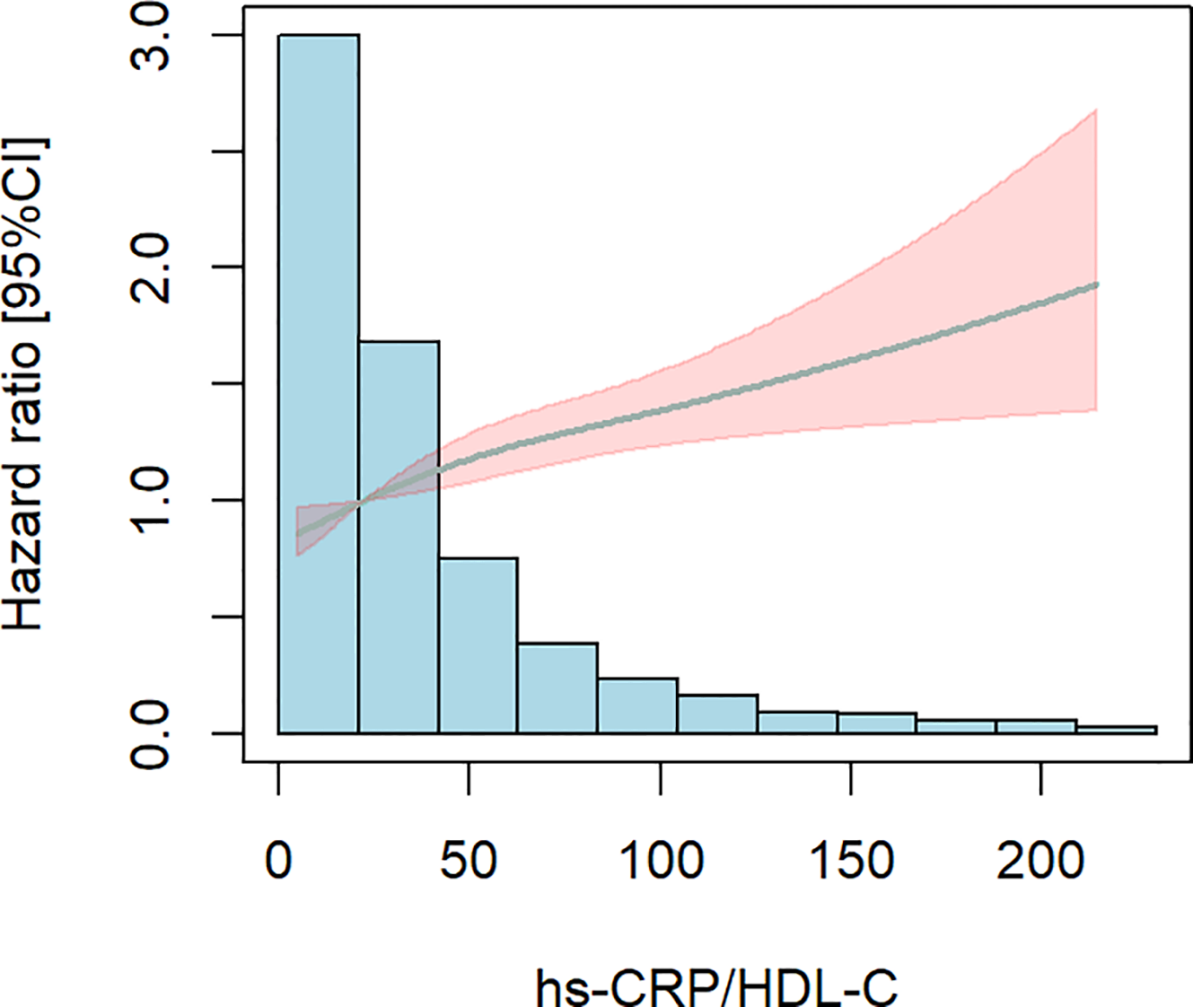
Limited cubic splines (RCS). The shaded area is specific to age, gender, marital status BMI,The smoking and drinking situation, hypertension, diabetes, and dyslipidemia were adjusted. There is no non-linear correlation. P for total< 0.001, P for nonlinear=0.187.

Subsequently, we used limited cubic splines (RCS) to ascertain the presence of a nonlinear relationship between the hs-CRP/HDL-C ratio and all-cause mortality in people with CKM stages 1-4. **Figure 4** illustrates the absence of a nonlinear relationship between the hs-CRP/HDL-C ratio and all-cause mortality in patients with CKM stages 1-4 (P for total < 0.001, P for nonlinear = 0.187). An elevation in the hs-CRP/HDL-C ratio correlated with a notable increase in all-cause mortality among persons with CKM stages 1-4.

**Figure 5:**
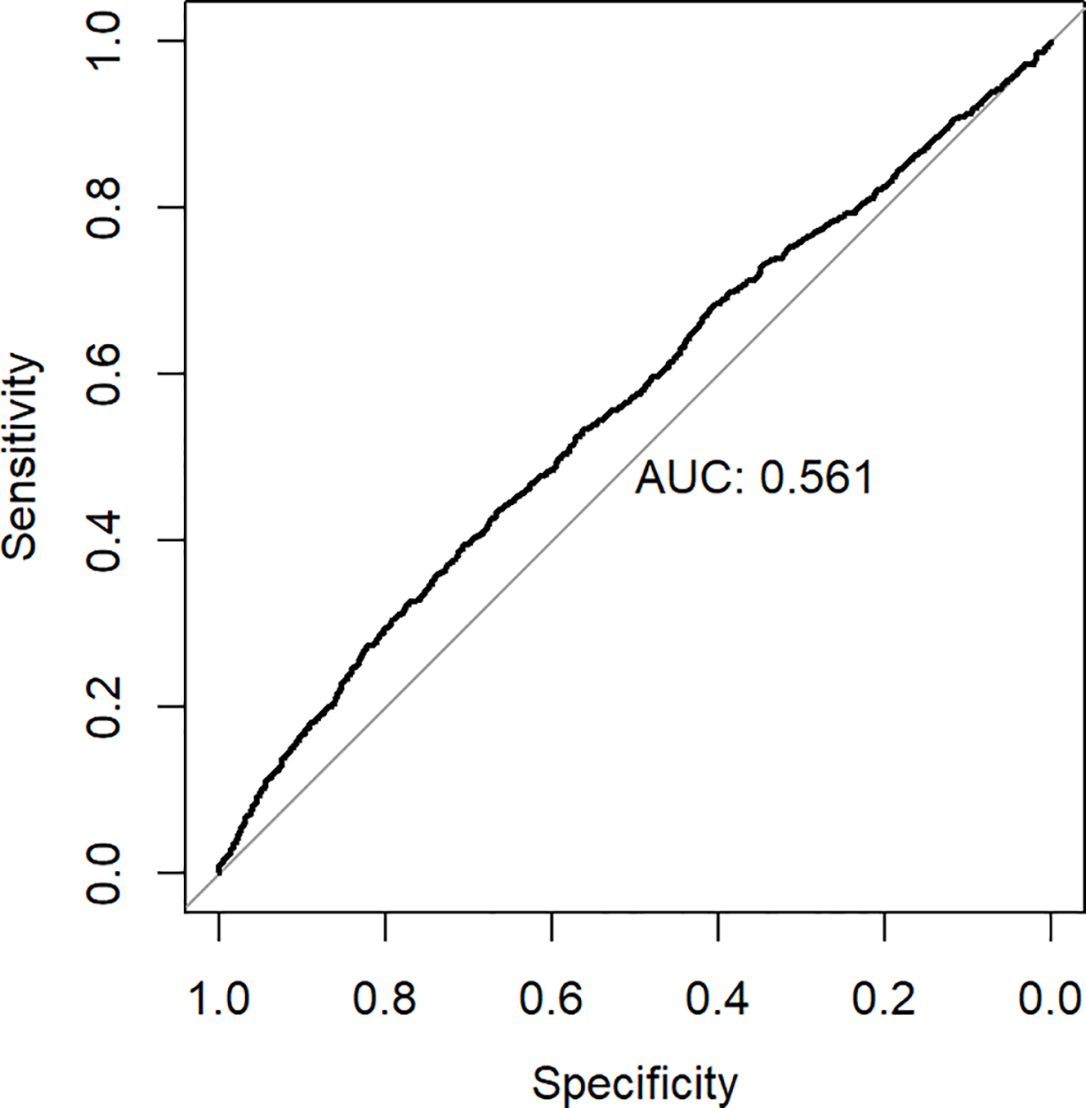
Receiver operating curve for hs-CRP/HDL. AUC,Area under the curve.

**Figure 6:**
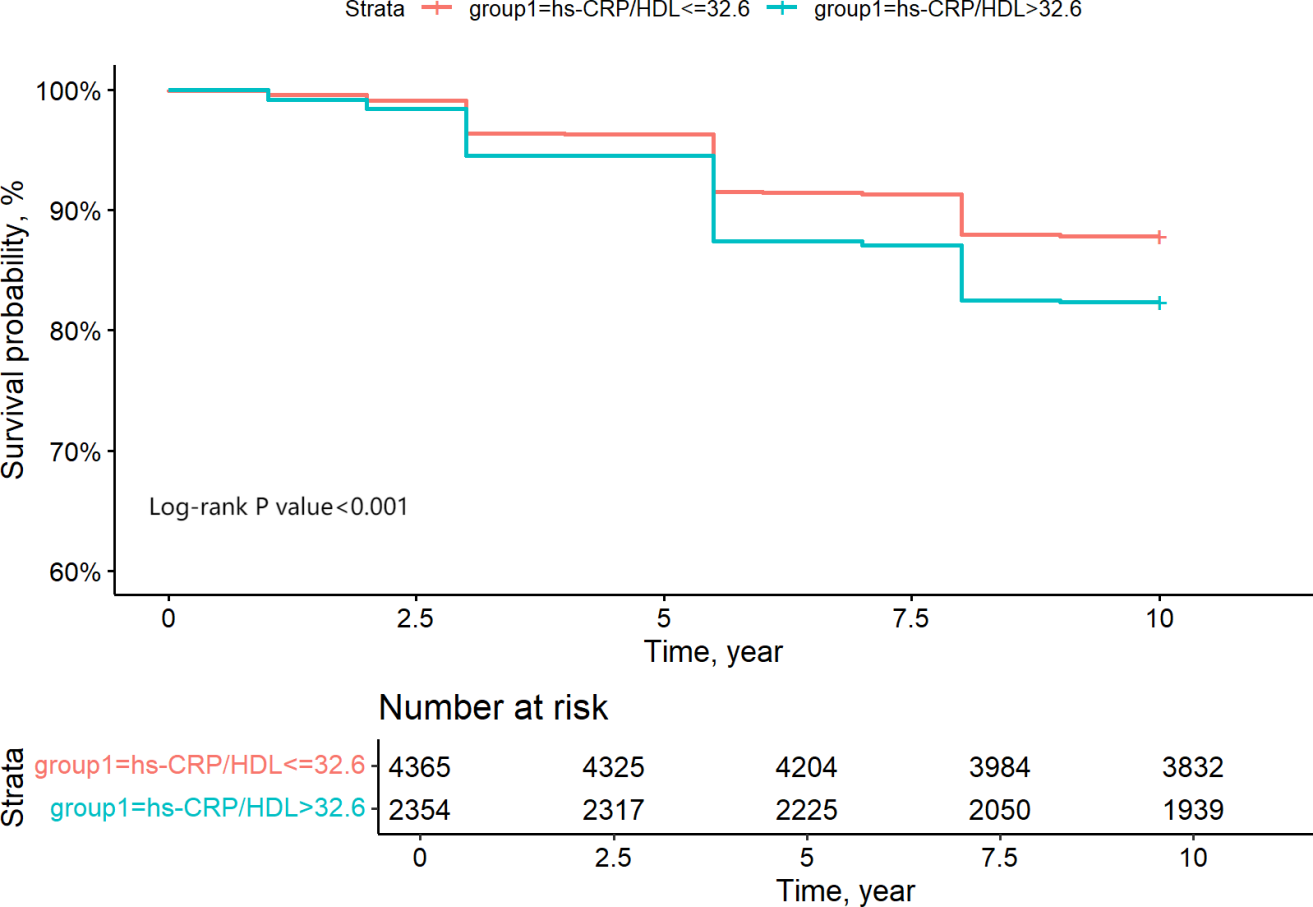
Grouping of CKM1-4 participants based on ROC analysis of the binary indicator of the optimal critical value of 10-year survival rate for hs-CRP/HDL quartiles.

ROC analysis was employed to ascertain the appropriate cut-off value of the hs-CRP/HDL-C ratio for predicting the risk of all-cause death. The research determined an appropriate cut-off value of 32.6, exhibiting a sensitivity of 43.8% and a specificity of 66.4%. **Figure 5** illustrates that the area under the curve (AUC) was 0.561 (95% CI 0.541-0.582). The hs-CRP/HDL-C ratio, utilised as a dichotomous indicator according to the optimal cut-off value for all-cause mortality established through ROC analysis, revealed that the 10-year survival rate for patients with a high hs-CRP/HDL-C ratio was markedly inferior to that of patients with a low hs-CRP/HDL-C ratio, as illustrated by the Kaplan-Meier survival curve in **Figure 6** (Log-rank test P-value < 0.001). The baseline risk model forecasting the 10-year all-cause mortality risk in patients with stages 1-4 CKM demonstrated exceptional concordance between observed and anticipated probabilities (see to **Supplementary Figure S3** for details). **Table 3** illustrates that the inclusion of the hs-CRP/HDL-C ratio in the baseline risk model markedly enhanced its predictive capability, as seen by an increase in the C-statistic from 0.753 to 0.760 (P = 0.032). This outcome significantly underscores the relevance of the hs-CRP/HDL-C ratio in prognostication. Furthermore, the integration of the hs-CRP/HDL-C ratio into the baseline risk model resulted in notable improvements in net reclassification improvement (NRI) and integrated discrimination improvement (IDI) (P < 0.05), thereby reinforcing the significance of the hs-CRP/HDL-C ratio in forecasting the risk of all-cause mortality in individuals with stages 1-4 CKM.

**Table 3:** Added predictive ability and reclassification statistics of hs-CRP/HDL.

Variables	C-statistic (95% CI)	P value	IDI (95% CI)	P value	Continuous NRI (95% CI)	P value
Baseline risk model	0.753(0.719,0.787)	ref	ref	ref	ref	ref
+hs-CRP/HDL	0.760(0.726,0.794)	0.032	0.009(0.0004 - 0.017)	0.041	0.296 (0.155 - 0.437)	<0.001

Baseline risk model: age, gender, education, marital status, current smoking status, DM, BMI, Fasting blood glucose and blood uric acid.

**Figure 7:**
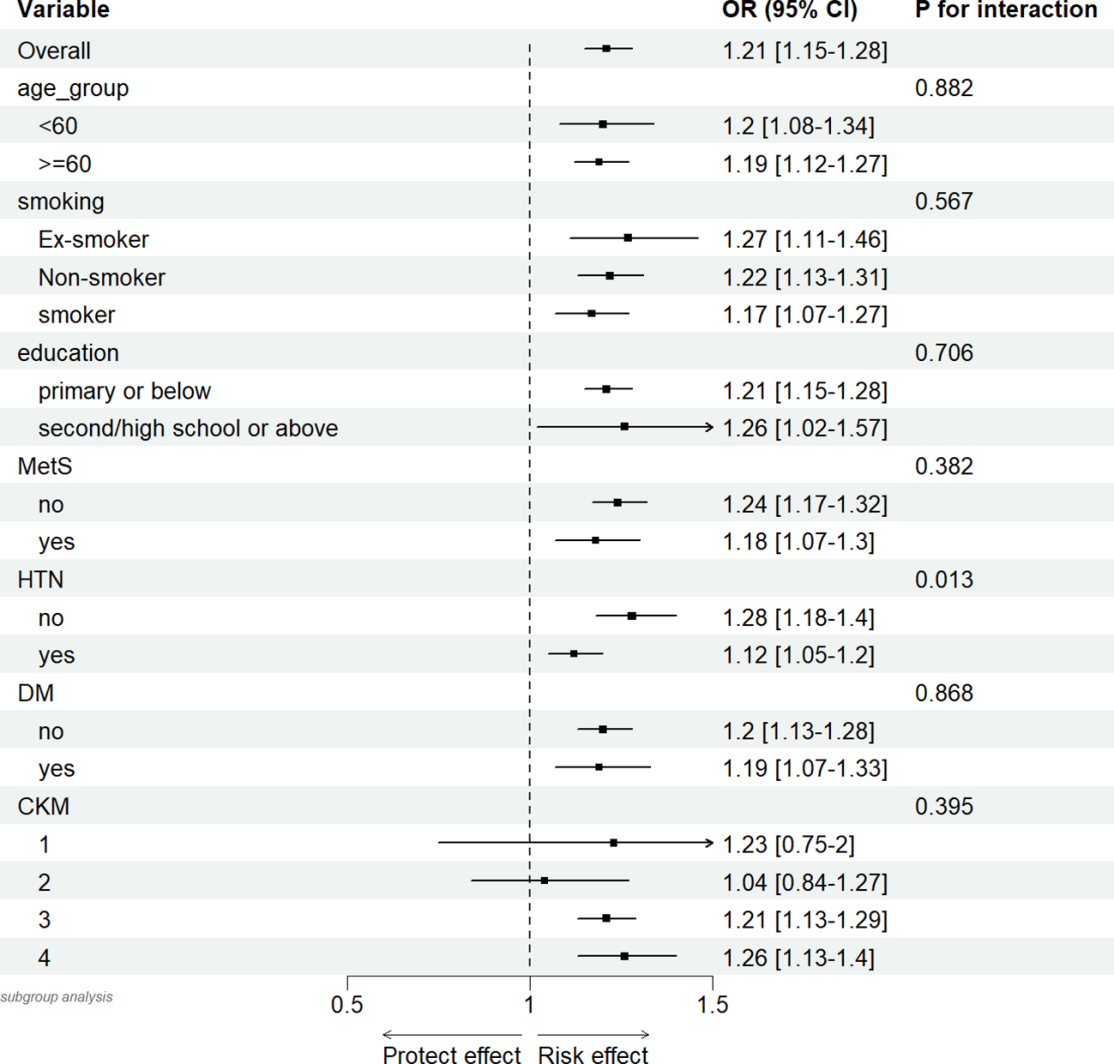
Association between hs-CRP/HDL (per quartile increase) in different subgroups and 10-year mortality risk in CKM syndrome stage 1-4 participants.

Finally, we conducted subgroup analyses and interaction analyses for age, smoking status, education level, DM, HTN, MetS, and CKM stages. As shown in the figures, the results (**Figure 7**) indicated that there was only an interaction between the presence or absence of hypertension (interaction P = 0.013). No interactions were observed in other subgroups (interaction P > 0.05).

## Discussion

This research is a prospective cohort study involving 6,719 eligible people, investigating the potential relationship between the hs-CRP/HDL-C ratio and the risk of all-cause death within the framework of CKM syndrome for the first time. The study findings indicated a substantial positive association between the hs-CRP/HDL-C ratio and the probability of all-cause mortality in patients with stages 1-4 CKD. The Kaplan-Meier curves demonstrate that the survival rate of patients in the Q4 group is significantly lower than that of the other three groups. To further elucidate the dose-response relationship between the two, additional Cox regression analysis was conducted, revealing that for every increase in the hs-CRP/HDL-C ratio across one quartile, the risk of death increases by 14%. However, when considering the hs-CRP/HDL-C ratio as a categorical variable, statistically significant differences were only observed between Q1 and Q4. The Restricted Cubic Splines (RCS) curves confirmed the absence of a nonlinear relationship, leading us to conclude that there is a positive linear correlation between the hs-CRP/HDL-C ratio and all-cause mortality. ROC curve analysis revealed an appropriate cut-off value of 32.6 for the hs-CRP/HDL-C ratio. Moreover, our investigation revealed that the inclusion of the hs-CRP/HDL-C ratio as a variable in the baseline prediction model markedly enhanced the model’s accuracy in forecasting the probability of all-cause death in patients with stages 1-4 CKM.

The deterioration of Cardiovascular-Kidney-Metabolic (CKM) syndrome health status frequently heralds an elevated risk of premature mortality and heightened morbidity. The interplay between lipid metabolism and inflammatory responses plays a pivotal role in augmenting the burden of cardiovascular diseases (CVDs) and accelerating renal dysfunction. Disruptions in lipid metabolism exacerbate tubular injury and propel the progression of interstitial fibrosis. Notably, the resultant oxidized high-density lipoprotein (Ox-HDL) can induce proinflammatory pathways, encompassing the upregulation of tumor necrosis factor-alpha (TNF-α), CC motif chemokine 2, while also augmenting reactive oxygen species (ROS) generation and exerting direct toxic effects on the kidney parenchyma (34–36). Furthermore, the deterioration of CKM health status is largely attributable to the substantial burden of CVDs (1). The hs-CRP/HDL-C ratio, as a novel inflammatory-lipid complex index, has been confirmed by multiple studies to be an important biomarker for the occurrence and development of CVD. Yano et al. demonstrated a significant correlation between the ratio of high-density lipoprotein cholesterol to C-reactive protein and left ventricular diastolic function as well as right ventricular systolic function in patients with heart failure with preserved ejection fraction, suggesting that this ratio may serve as a predictor for all-cause and cardiogenic mortality risk in these individuals (37). A separate study indicated that an elevated hs-CRP/HDL-C ratio serves as a significant independent risk factor for cardiovascular disease, stroke incidence, and cardiac issues (28). The hs-CRP/HDL-C ratio is substantially correlated with adverse clinical outcomes in patients with acute ischaemic stroke (38).

Hypersensitive C-reactive protein (hs-CRP), traditionally regarded as a reactant in the acute phase of inflammation and mainly synthesized in hepatocytes, has increasingly been shown in recent studies to also express its mRNA and protein in arterial plaque tissue, with levels 10 times higher than in normal arterial tissue, resulting in locally much higher concentrations of hs-CRP in plaques compared to those in plasma. Locally in the arteries, hs-CRP not only inhibits the release of nitric oxide (NO) and vascular dilation but also enhances NADPH oxidase activity in the arterial endothelium, promoting the generation of superoxide and subsequently triggering endothelial dysfunction, thereby exerting pro-inflammatory and pro-atherosclerotic effects (39). Furthermore, hs-CRP stimulates macrophages to secrete inflammatory cytokines such as interleukin-8 (IL-8), interleukin-6 (IL-6), and tumor necrosis factor (TNF) (40). Among these, IL-6 has been shown to upregulate the expression of cell adhesion molecules, leading to persistent damage to endothelial barrier function (41).

Conversely, high-density lipoprotein (HDL) serves several protective functions in atherosclerosis prevention. HDL significantly mitigates the onset and progression of atherosclerosis by reversing cholesterol transport, displaying antioxidant properties, demonstrating anti-inflammatory actions, providing anti-thrombotic effects, and enhancing endothelial function (42, 43). The antioxidant properties of HDL primarily rely on its protein constituents, apolipoprotein AI (apo AI) and paraoxonase 1 (arylesterase, EC 3.1.8.1). Paraoxonase 1, an enzyme carried by HDL components, prevents the oxidative alteration of low-density lipoprotein (LDL), hence obstructing a crucial step in atherosclerosis formation (44, 45).

However, recent research has revealed that HDL particles are highly heterogeneous. Normal HDL (nHDL) exhibits a positive promotional effect on angiogenesis, emphasizing its role in maintaining vascular health. In contrast, HDL present in patients with coronary artery disease (CAD), termed dysfunctional HDL (dHDL), exhibits a dysfunctional state that not only fails to perform its normal physiological functions but also impairs angiogenesis. This finding challenges the traditional view that HDL solely possesses anti-atherosclerotic properties, revealing the dual nature of HDL in the atherosclerotic process—both potentially pro-atherosclerotic and anti-atherosclerotic. These studies also suggest that simply increasing HDL levels is not sufficient to ensure a reduction in cardiovascular disease risk (46, 47). They highlight the complex roles of hs-CRP and HDL in the pathophysiology of atherosclerosis, providing new insights into understanding the pathogenesis of CKM syndrome.

Hypersensitive C-reactive protein (hs-CRP) and high-density lipoprotein cholesterol (HDL-C) are regarded as critical biomarkers of inflammation and endothelial dysfunction, respectively, serving opposing functions in the modulation of inflammatory responses—hs-CRP facilitates inflammation, whereas HDL-C may suppress it. These two biomarkers collaboratively engage in the pathophysiological mechanisms that contribute to the emergence of cardiorenal-metabolic (CKM) syndrome, indicating that this inflammatory-lipid complex may serve as a significant predictor of long-term mortality risk in patients with CKM syndrome. Our study confirms this assertion, demonstrating that the long-term mortality risk in patients with CKM syndrome, increases significantly with elevated hs-CRP/HDL-C ratios, exhibiting a positive linear correlation between the two. We hypothesize that this association may stem from the following two factors: Firstly, as an inflammatory marker, elevated hs-CRP levels reflect heightened inflammatory responses in the body, which may further exacerbate renal damage and facilitate the progression of cardiovascular diseases. Secondly, High-Density Lipoprotein (HDL) plays a crucial role in reverse cholesterol transport, aiding in the removal of excess cholesterol from peripheral tissues back to the liver for metabolism and excretion. However, in patients with CKM syndrome, HDL functionality is often impaired, resulting in decreased cholesterol transport capacity and subsequent lipid accumulation, which may accelerate renal function deterioration and promote atherosclerosis development. Consequently, an increased hs-CRP/HDL-C ratio may indicate intensified inflammatory responses and lipid accumulation in the body, thereby elevating the long-term all-cause mortality risk in patients with CKM syndrome stages 1-4. This study not only solidifies the hs-CRP/HDL-C ratio’s role as a predictive factor but also demonstrates its capacity to enhance the precision of clinical prognostic predictions. Thus, the hs-CRP/HDL-C ratio may serve as a significant biomarker for identifying high-risk individuals and aiding therapeutic decision-making, offering new insights for risk stratification and personalized therapy of CKM syndrome patients.

The strengths of our research are outlined below: Firstly, our study is a prospective cohort study with a large sample size, distinguished by its systematic assessment of the association between the hs-CRP/HDL-C ratio and long-term adverse prognosis in CKM syndrome patients, and an in-depth exploration of the specific manifestations of this association. This innovative aspect not only fills a research gap in this field but also provides important reference material for subsequent studies. Secondly, in terms of study design, we analyzed the hs-CRP/HDL-C ratio both as a categorical variable (divided by quartiles) and as a continuous variable to comprehensively evaluate its association with the risk of all-cause mortality. This analytical strategy not only helps to identify risk differences at different levels of hs-CRP/HDL-C but also better reflects clinical realities. Thirdly, hs-CRP and HDL-C, as test indicators, have the advantages of simple and easy detection, accessibility, and relatively low cost. These characteristics make them highly valuable in clinical practice, especially in primary health care. Lastly, to ensure the credibility of our results, we conducted sensitivity studies, which confirmed the reliability of our research.

However, this study also has certain limitations. Firstly, our research is focused solely on middle-aged and elderly individuals over 45 years old in China, and additional validation is needed to assess the generalizability of these findings to other racial groups and age groups. Secondly, the diagnosis of chronic diseases relies on self-reporting by subjects, which may introduce bias. Thirdly, the CHARLS dataset has room for improvement in recording the causes of patient deaths. We only considered all-cause mortality among patients and lacked research on the risk of cardiovascular death.

## Conclusion

This cohort study demonstrates a substantial linear relationship between the hs-CRP/HDL-C ratio and long-term all-cause mortality in patients with stages 1-4 of CKM syndrome. This discovery indicates that a comprehensive evaluation of the hs-CRP/HDL-C ratio may serve as a more effective and efficient screening instrument for identifying high-risk patients with stages 1-4 of CKM syndrome. Moreover, as an easily obtainable and economical biomarker, this ratio exhibits significant potential in prognostic evaluation.

## Data Availability

The datasets presented in this study can be found in online repositories. The names of the repository/repositories and accession number(s) can be found below: http://charls.pku.edu.cn.
