# Supplementary material for: hs-CRP/HDL-C can predict the risk of all cause mortality in cardiovascular-kidney-metabolic syndrome stage 1-4 patients
Source: Front Endocrinol (Lausanne). 2025 Apr 10;16:1552219. doi: 10.3389/fendo.2025.1552219 (PMC12018248; doi:10.3389/fendo.2025.1552219)
Supplement: Supplementary file 1 [file DataSheet1.docx]

| P值 | Q1 | Q2 | Q3 | Q4 |
| --- | --- | --- | --- | --- |
| Q1 | - |  |  |  |
| Q2 | 0.7 | - |  |  |
| Q3 | 0.2 | 0.4 | - |  |
| Q4 | <0.001 | <0.001 | <0.001 | - |

**Supplementary Table S1** Kapla-Meier analysis of P-values compared between different groups

**Supplementary Figure S1:**Flowchart of the study population


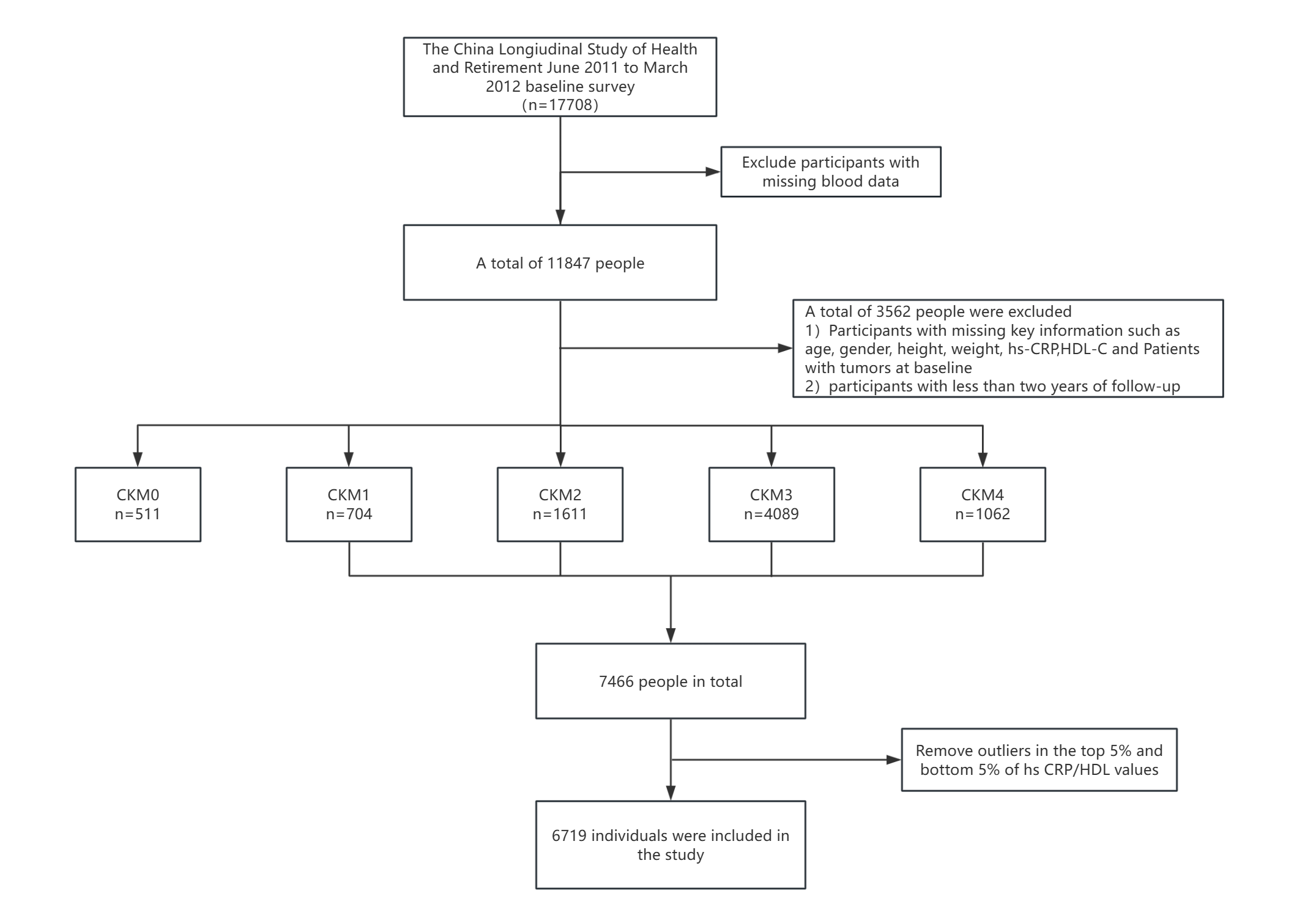


**Supplementary Figure S2:** Kapla-Meier analysis after reintroducing participants with hs CRP/HDL values in the top 5% and bottom 5%


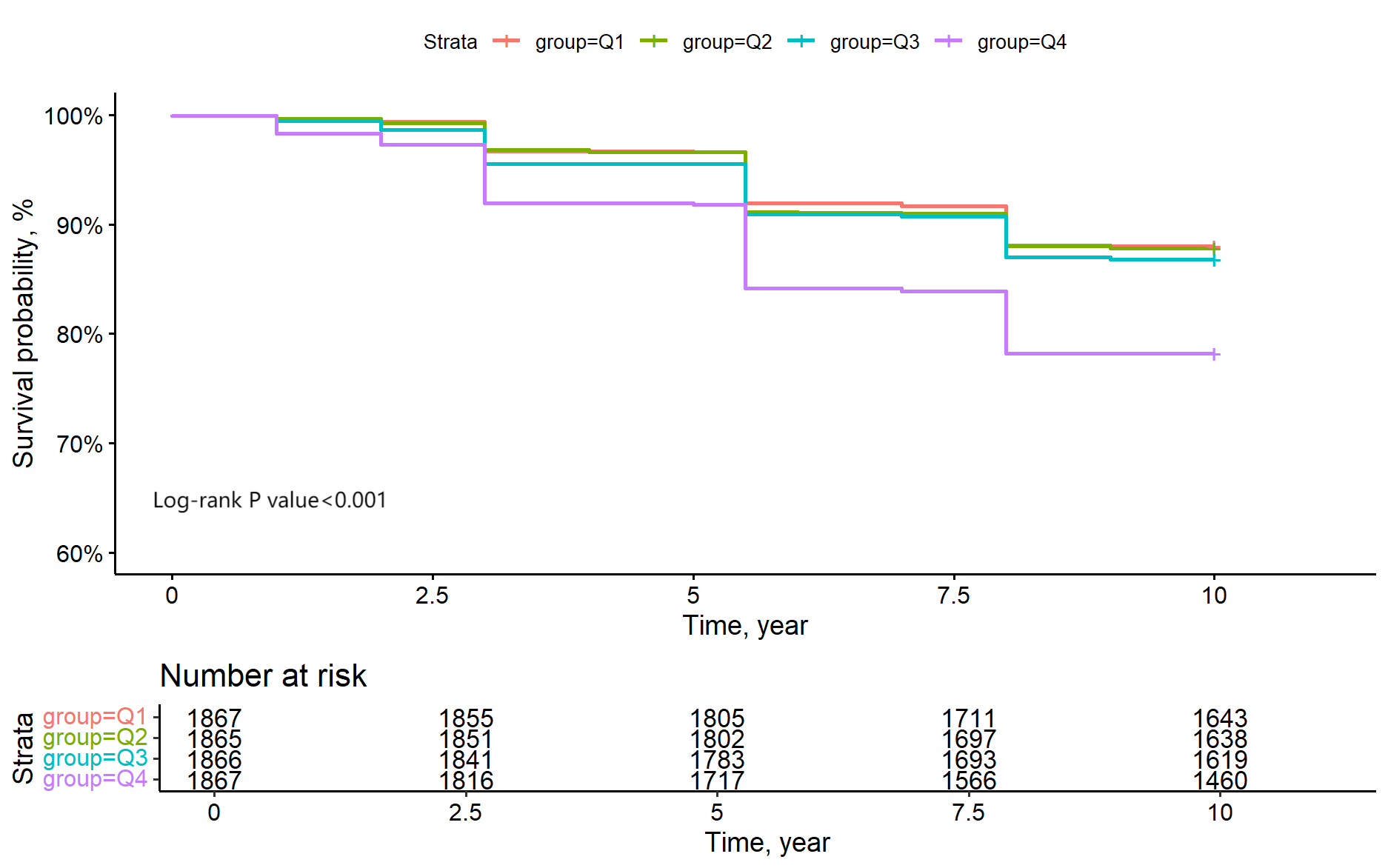


**Supplementary Table S2:** Cox regression analysis after reintroducing participants with hs CRP/HDL values in the top 5% and bottom 5%

| Variables | Model 1 | | Model 2 | | | Model 3 | | |
| --- | --- | --- | --- | --- | --- | --- | --- | --- |
|  | HR(95%) | P value | HR(95%) | P value | | HR(95%) | P value | |
| hs-CRP/HDL per IQR | 1.03(1.02,1.03) | <0.001 | 1.02(1.01,1.03) | <0.001 | | 1.02(1.01,1.03) | <0.001 | |
| hs-CRP/HDL quartile |  | | | | | | | |
| Q1 | ref | | ref | | | ref | | |
| Q2 | 0.97(0.81,1.17) | 0.77 | 1.01(0.81,1.25) | | 0.95 | 1.01(0.81,1.27) | | 0.89 |
| Q3 | 1.05(0.87,1.26) | 0.61 | 1.19(0.96,1.47) | | 0.11 | 1.18(0.95,1.47) | | 0.14 |
| Q4 | 1.65(1.40,1.95) | <0.001 | 1.85(1.52,2.25) | | <0.001 | 1.78(1.44,2.20) | | <0.001 |

Model 1: adjusted age,gender

Model 2:adjusted age,gender,marital,bmi,smoking,drinking,education,Annual household income and Lives in rural or urban

Model 3:age,gender,marital,bmi,smoking,drinking,education,Annual household income,Lives in rural or urban,DM,HTN,medication history,triglycerides ,glucose ,ldl cholesterol ,uric acid ,total cholesterol and creatinine

**Supplementary Figure S3:** Calibration chart of the adjusted model for predicting the 10-year mortality risk of CKM syndrome stages 1-4.


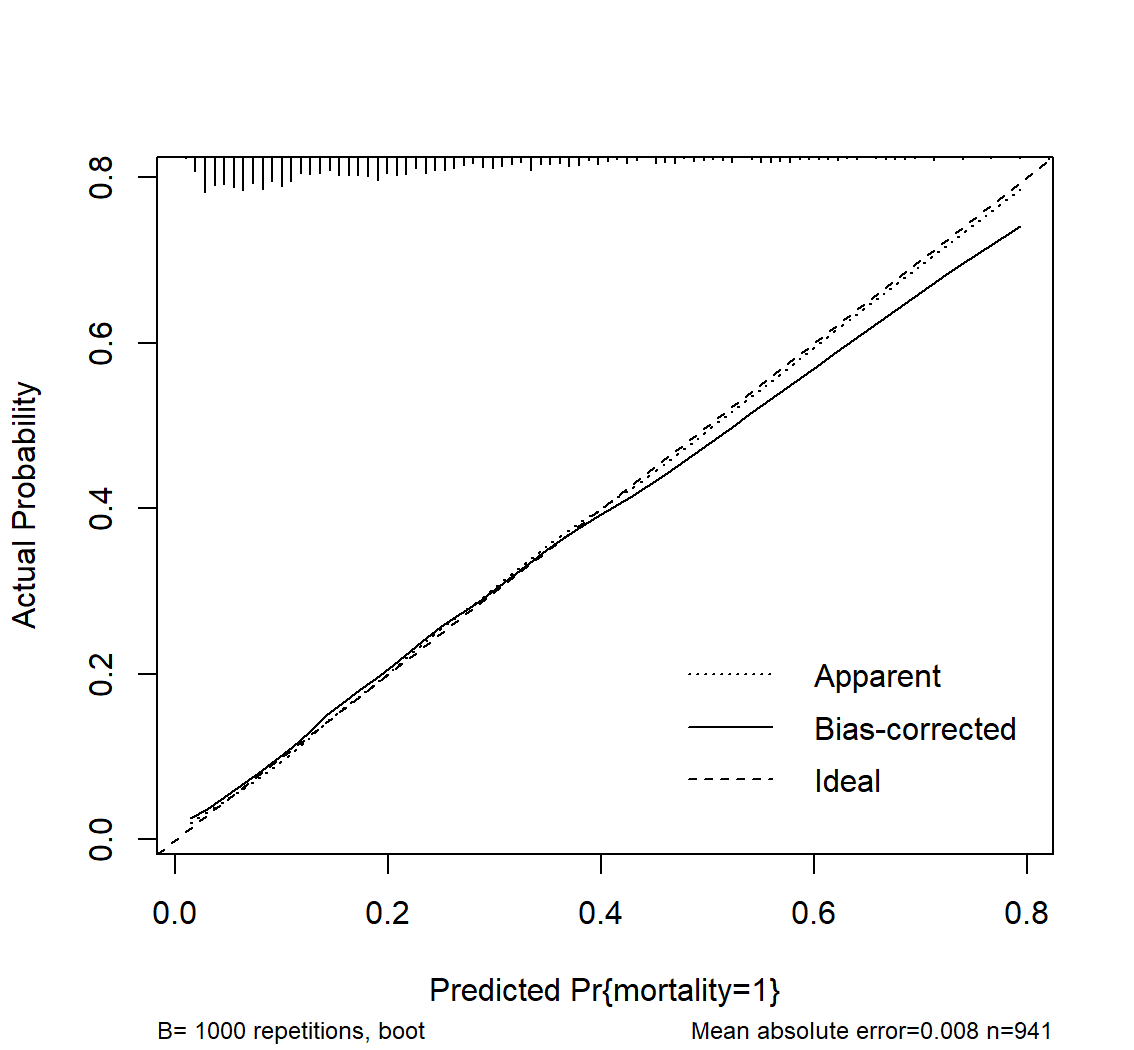


The x-axis represents the predicted risk of death. The y-axis represents the actual mortality rate. The dashed line represents the perfect prediction of the ideal model. The solid line represents the performance of the prediction model, where the closer the fit to the dashed line, the better the prediction effect. The adjusted model refers to the established basic risk model.The basic risk model established includes age, gender, education level, marital , current smoking,DM,BMI,Fasting blood glucose, and uric acid.
